# Digital PCR: modern solution to parasite diagnostics and population trait genetics

**DOI:** 10.1186/s13071-023-05756-7

**Published:** 2023-04-25

**Authors:** Paulius Baltrušis, Johan Höglund

**Affiliations:** Department of Biomedical Sciences and Veterinary Public Health, Section for Parasitology, Swedish University of Agricultural Sciences, Uppsala, Sweden

**Keywords:** ddPCR, Diagnosis, Parasite, Resistance, eDNA

## Abstract

The use of polymerase chain reaction (PCR)-based diagnostic approaches has steadily increased in the field of parasitology in recent decades. The most recent large-scale technological modification of the PCR formula, also known as third-generation PCR, came in the form of digital PCR (dPCR). Currently, the most common form of dPCR on the market is digital droplet PCR (ddPCR). Unlike quantitative real-time PCR (qPCR), the digital format allows for highly sensitive, absolute quantification of nucleic acid targets and does not require external standards to be included in the developed assays. Dividing each sample into thousands of compartments and using statistical models also eliminates the need for technical replicates. With unprecedented sensitivity and enforcement of binary endpoint reactions, ddPCR not only allows the use of tiny sample volumes (especially important when working with limited amounts of DNA) but also minimises the impact of variations in amplification efficiency and the presence of inhibitors. As ddPCR is characterised by excellent features such as high throughput, sensitivity and robust quantification, it is widely used as a diagnostic tool in clinical microbiology. Due to recent advances, both the theoretical background and the practical, current applications related to the quantification of nucleic acids of eukaryotic parasites need to be updated. In this review, we present the basics of this technology (particularly useful for new users) and consolidate recent advances in the field with a focus on applications to the study of helminths and protozoan parasites.

**Graphical Abstract:**

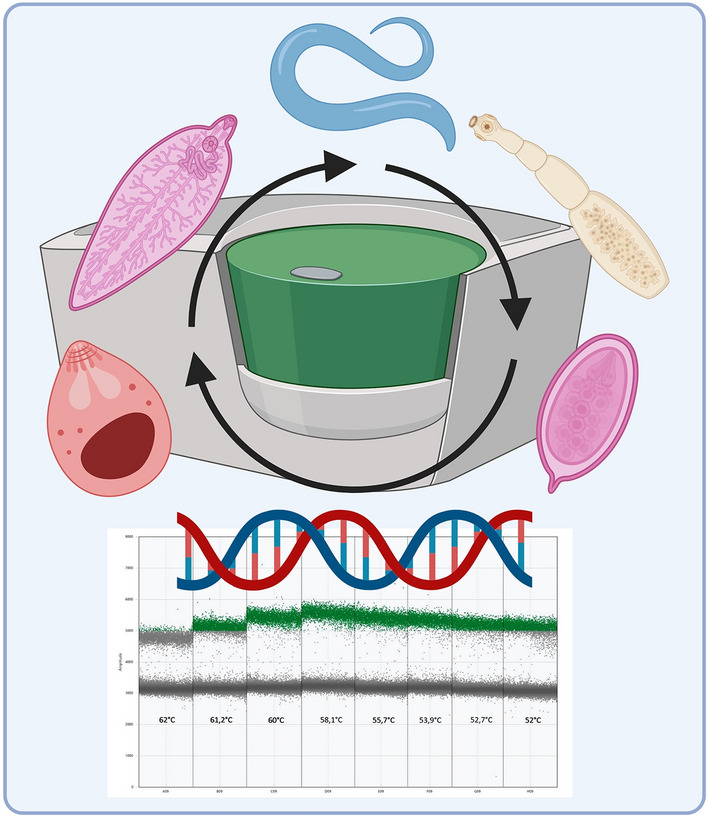

## Background

Parasites, some more pathogenic than others, are still abundant and cause disease and discomfort in animals and humans. Despite major advances by the pharmaceutical industry in the development of drugs against parasites, parasitic infections are widespread and represent a significant burden of disease in many areas of the world. For example, according to the relatively recent Pan-European Economic Assessment, the cost of helminth infections in ruminants is estimated to be similar to or higher than the cost of animal diseases and has been estimated at €941 million per year [1]. The impact of neglected parasitic diseases on human society, especially on children in resource-poor countries, is also immense [2]. To make matters worse, zoonotic parasites can circulate between different hosts and, in combination, pose a major disease burden for those infected through contact with contaminated food, water, soil or via vectors [3, 4].

Understanding the composition of parasite communities and their impact on disease risk is critical to any surveillance programme focused on reducing the parasite burden. Similarly, having the most up-to-date diagnostic tools can enable more efficient use of resources for parasite control. For decades, parasitologists have routinely relied on traditional diagnostic techniques based on microscopic examination. While such techniques are simple and inexpensive, they have problems with reproducibility and are often not specific and sensitive enough. In addition, microscopic methods are usually labour-intensive and require experienced staff to perform them. The latter is particularly important as we are likely to face a shortage of trained professionals with the skills and expertise required to identify parasites the old-fashioned way as we move into the ‘molecular tool-based future’.

The introduction of nucleic acid-based detection methods may not only lead to more accurate diagnosis but also contribute to a more efficient and unbiased screening of parasites, not least given the potential for integrating complementary assays into highly automated platforms. By refining diagnostics, we are likely to be able to assign more effective antiparasitic treatments, which is of paramount importance given the ever-increasing number of cases of resistance to one or more types of drugs. The said problem is particularly evident in helminths of grazing livestock [5] but is also of concern in the soil-transmitted human helminths [6], and protozoa such as *Plasmodium* [7]. The development of rapid and sensitive high-throughput tools that can (Table 1) detect and quantify parasites and determine their resistance status with high accuracy is therefore crucial.

**Table 1:** ddPCR applications in parasitology, sorted by assay type

Parasite	Sample	Target	Assay	Detection	Purpose	Refs.
*Cooperia, Ostertagia*	L3	ITS2	Discrimination	FAM/HEX	PD	[41]
*Haemonchus contortus*	L3	*β-tubulin* (F200Y)	Discrimination	FAM/HEX	AR	[38–40]
*Ascaridia* and* Heterakis*	Eggs in faeces	ITS2	Duplex	FAM/HEX	PD	[29]
*Eimeria* spp.	Oocysts	*cox3*	Duplex	FAM/HEX	PD	[30]
*Haemonchus contortus*	L3	*acr-8*	Duplex	FAM/HEX	PD	[35]
*Haemonchus contortus*	L3	*dyf-7*	Duplex	FAM/HEX	AR	[37]
*Haemonchus*, *Teladorsagia*, *Trichostrongylys*	L3	ITS2	Duplex	FAM/HEX	PD	[24–28]
*Plasmodium falciparum*	Blood	*hrp2*, *hrp3 tRNA*	Duplex	FAM/HEX	PD	[31]
*Plasmodium* spp.	Blood	18S RNA	Duplex	FAM/VIC		[32]
*Babesia*, *Bartonella*, *Borrelia*, *Theileria*	Blood, vectors	ITS	Duplex, multiplex	CY5.5	PD	[33]
*Plasmodium falciparum*	Mosquitos	*mdr1*, *plasmepsin2*, *ghc1*, *β-tubulin*	Duplex, multiplex	FAM/HEX	PD, IR	[36]
*Chilodonella hexasticha*	Water	SSU-rDNA	eDNA		PD	[42]
*Gyrodactylis salaris*	Water	ITS2, *cytb*	eDNA, duplex		PD	[43]
*Fasciola*	Water		eDNA, duplex		PD	[46]
*Taenia solium*	Soil	*cox1*	eDNA, uniplex	FAM	PD	[44, 45]
*Cytauxzoon felis*	Blood	*cox3*	Uniplex	Probe*	PD	[23]
*Dirofilaria immitis*	Microfilariae, blood	*nhr-7, nhr-6*	Uniplex	EvaGreen	Other	[34]
*Echinococcus multilocularis*	Liver	cob	Uniplex	Probe*	PD	[20]
*Haemosporidians*	Blood	*rRNA*	Uniplex	EvaGreen	PD	[22]
*Schistosoma japonicum*	Sample	*nad1*	Uniplex	EvaGreen	PD	[19]
*Toxoplasma gondii*	Diaphragm	Toxo-529 repeat	Uniplex		PD	[21]
*Trichuris* spp.	Worms	ITS1	Uniplex	Probe*	PD	[18]

*AR* anthelmintic resistance, *cytb* cytochrome b, *IR* insecticidal resistance, *PD* parasite detection, Probe* fluorophore not mentioned, *SSU* small subunit, *cox* cytochrome c oxidase genes, *cob* cythochrome b genes, *mdr* multidrug resistant mutation genes, *nhr* Nuclear hormone receptor genes

One of the most advanced methods that can do just this is ddPCR. While reviews of its applications have been published previously, these focused exclusively on human parasites [8, 9]. Since then, great progress has been made, and both the number of studies and the applications have greatly expanded (Table 1). In this review, we provide an up-to-date overview of the different reaction approaches available (uniplex, multiplex and discrimination tests) and how each of these approaches can be used not only to detect the major parasites in a variety of hosts but also to detect their resistance status to antiparasitic drugs. We also cover some examples of how the technology can be used to screen environmental samples (containing parasites). Our overall aim is to enable new users to get started and to stimulate wider application of digital PCR (dPCR) technology in parasitological research.

## Methods

### Search strategy

The records were identified through a search of international peer-reviewed literature. General search terms were used consisting of combinations of technology (e.g. dPCR or ddPCR) and type of parasite (helminth, nematode, protozoan, etc.). Titles were then identified by searching Google Scholar, PubMed, Web of Science and Scopus up to October 2022, focusing on publications that contained original information on the quantification of parasitic organisms and/or genetic variants associated with different traits. In the second step, the titles found were selected based on the information in the abstracts and full texts. Of the titles identified, 28 provided information that had been published since 2017 and generally had not been previously covered by any review.

## Results and discussion

### The technology

**Fig. 1:**
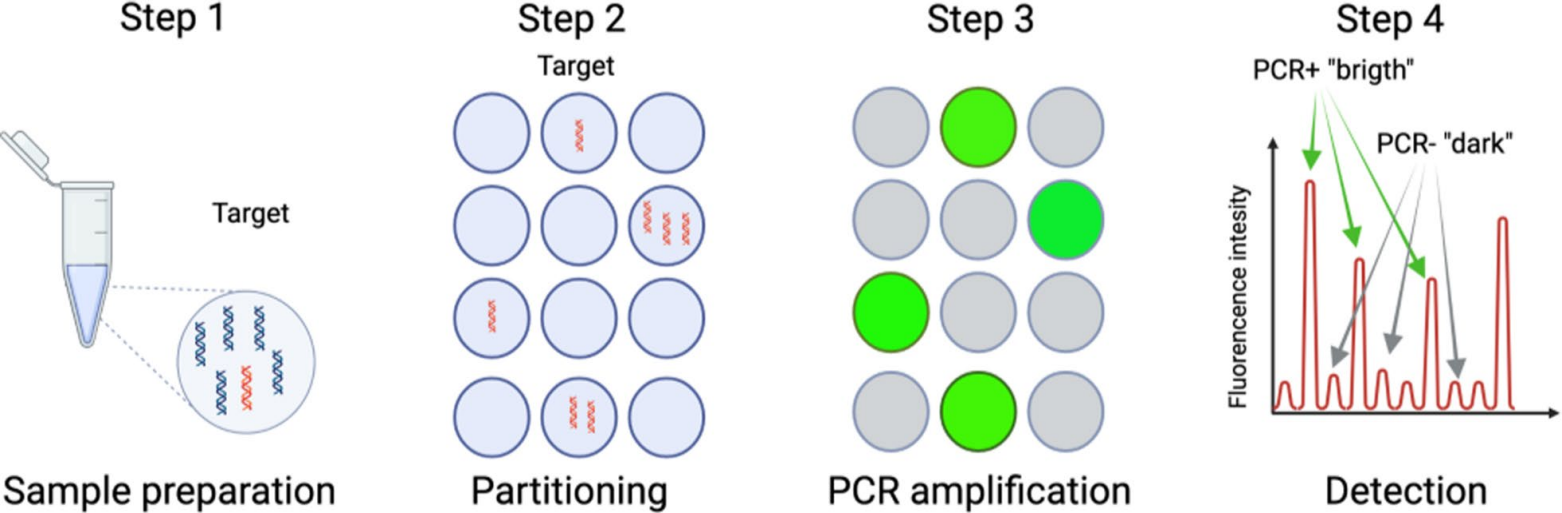
The workflow in ddPCR (the most commonly used platform in parasitology) can be divided into four steps. Step 1: A mixture of primers and DNA-binding dyes (such as EvaGreen^®^) or fluorescent probes (containing dye molecules such as FAM™, HEX™ or VIC^®^) at their 5′ end and a quencher molecule containing probes at their 3′ end is often mixed with an already prepared supermix (containing premixed amounts of DNA polymerase, deoxynucleoside triphosphates [dNTPs] and salt). Step 2: After sample preparation, microfluidics is used to disperse and subdivide each sample into several thousand nano-sized droplets (partitions), each containing either no template molecules or one or more template molecules of interest. Step 3: The target nucleic acid sequence(s) are subjected to conventional PCR amplification until an endpoint is reached. Step 4: The presence of PCR-positive and PCR-negative reactions is evaluated using a droplet reader (strictly binary). From this, the concentration of the analyte molecule in the sample can be determined by fitting a Poisson distribution to the data. Essentially, the relationship between the proportion of positive reactions and the copies of the target molecule (i.e. ln(1−p), where p is the proportion of positive reactions) is used to determine the copies of the target molecule (with a 95% confidence interval) in the volume of the sample-reaction mixture

Digital PCR technologies are a greatly improved version of conventional PCR (Fig. 1), allowing absolute quantification of target nucleic acids in a sample. The preparation of the reaction setup bears much resemblance to other commonly used types of PCR. However, unlike quantitative real-time PCR (qPCR), for example, where amplicon enrichment is reported in real time, the digital format relies on endpoint quantification after sample division [10]. The general principles as well as the technical advantages have been described in detail elsewhere [11, 12]. For each sample run, dPCR provides not only the exact analyte concentrations in the original sample (conveniently expressed in copies per microlitre) but also the confidence interval estimates, effectively eliminating the need for technical replicates or standard curves [12, 13]. In addition, partitioning the sample(s) ensures that different PCR inhibitors are diluted, resulting in better overall performance when working with chemically impure or otherwise ‘difficult’ samples [14]. To date, several companies including Bio-Rad, Fluidigm, Thermo Fisher and Qiagen offer both the necessary equipment (along with reagents) and basic guidelines for performing reactions properly. Perhaps the most common form of dPCR at present is ddPCR, in which the partition step involves dispersing the sample into thousands of water-in-oil droplets (e.g. Bio-Rad and Stilla). Other forms of dPCR perform the sample partitioning step with physical arrays instead (e.g. Fluidigm, Thermo Fisher and Qiagen) [15]. To date, almost all dPCR assays used to study eukaryotic parasites have used Bio-Rad's ddPCR platform.

### Types of assays

**Fig. 2:**
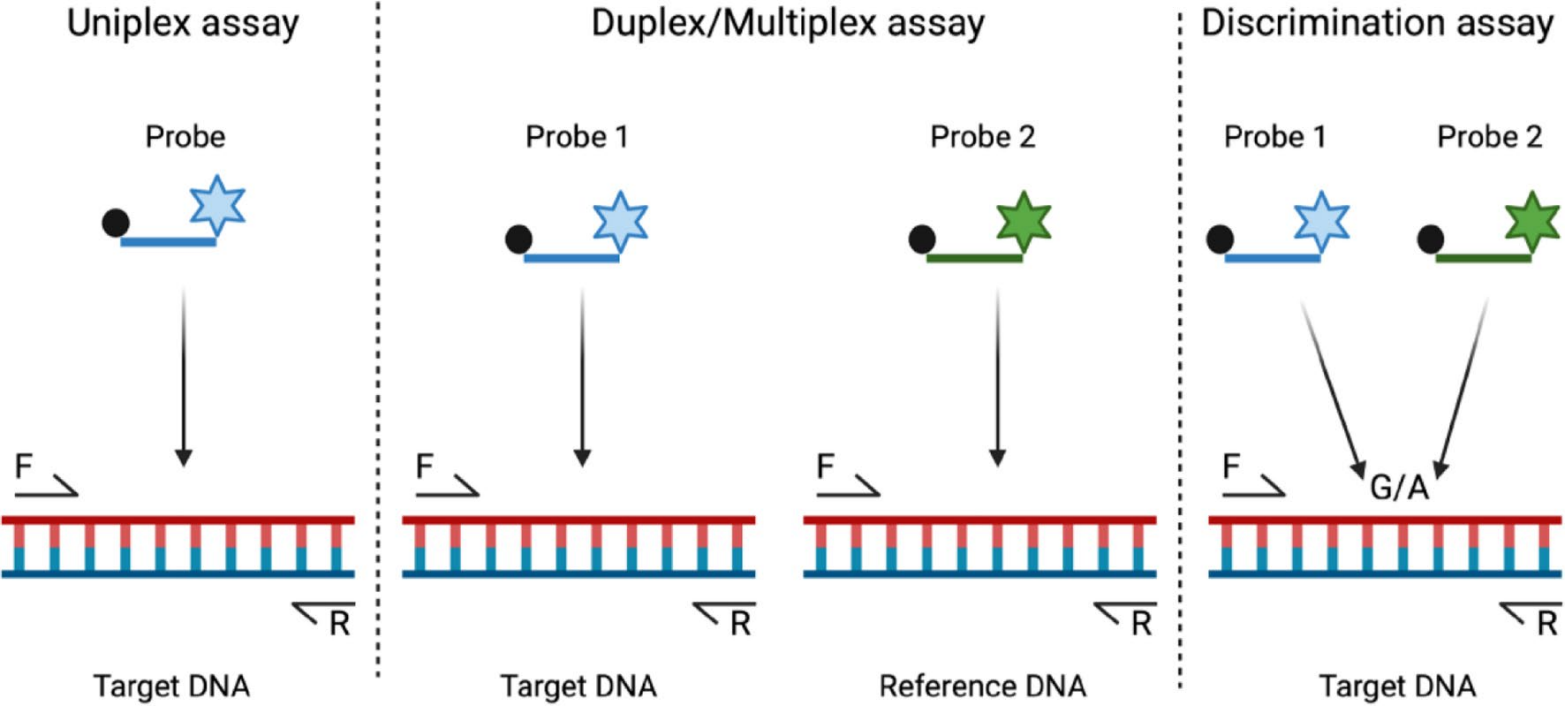
Three different assay types. The black dot represents the quencher of the fluorophore molecule (stars). F and R are the forward and reverse primers, respectively

Next, we will describe the three types of assays commonly used in the study of different parasites and their communities (Fig. 2). The simplest of the three assays—the uniplex (simplex) assay—relies on primer pair-mediated amplification of the target DNA sequence of interest using minute amounts of parasite material. In a typical scenario, the length of the amplicon fragment is usually chosen between 80 and 200 base pairs (bp), although amplicon length is less important than in qPCR. After endpoint amplification, detection in dPCR is based on either the inclusion of DNA-binding dyes (e.g. EvaGreen^®^) or a TaqMan hydrolysis probe. In a duplex or multiplex assay, at least two different primer pairs are used. Each amplified target sequence is detected with hydrolysis probes labelled with different fluorescent molecules that can emit fluorescence (at different wavelengths) when excited. Before attempting to perform this type of assay on actual samples, one should ensure that the different primer and probe sets do not interfere (i.e. cross-react) with each other. This can be easily determined by comparing the target copy numbers obtained for each primer–probe pair (for uniplex reactions) with those achieved with a duplex/multiplex setup using a (set of) reference sample(s). The third type, a discrimination test, facilitates the discrimination of sequence variants (e.g. single-nucleotide polymorphisms [SNPs], short indels) within the same amplicon by using two differently labelled, competing probes. Each of these probes is synthesised to reflect the sequence variant it is designed to selectively bind to and detect.

**Fig. 3:**
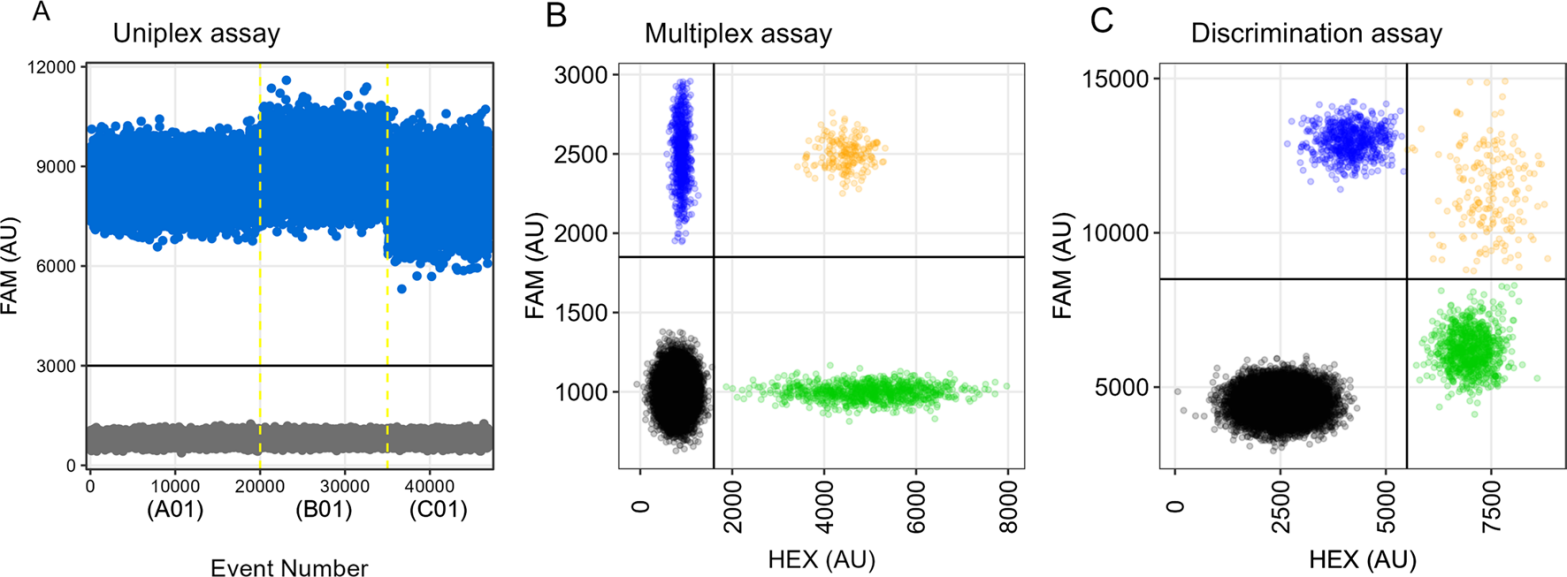
Examples of the results of uniplex (**A**), duplex (**B**) and discrimination tests (**C**). In a typical one-dimensional diagram for uniplex assays (**A**), the positive droplets (for fluorescence with a single fluorophore, e.g. FAM) are separated from the negative ones by an adjustable threshold (here a black line set at 3000 AU), while all droplets obtained per sample-containing well (e.g. A01) are separated from the others (i.e. B01) by vertical yellow dashed lines. Both the positive and negative controls and the amplitude of fluorescence (on the *y*-axis; usually expressed in AU) are used to distinguish the droplet populations into positive and negative. In a two-dimensional diagram (**B**), typically used in the analysis of fluorescence emitted by two (or more) fluorophores simultaneously, the amplitudes for the four droplet clusters (double negative—grey, FAM-positive—blue, HEX-positive—green and double positive—orange) and thus the composition of each sample in relation to the two DNA targets are determined by simultaneously setting thresholds on the x and y axes. In discrimination tests (**C**), which are duplex tests by default, the two-dimensional plots resemble those of other duplex tests. However, droplet clusters tend to align more closely (especially in tests based on SNP frequency estimation) due to the generally low, indiscriminate binding of probes that are similar in nucleotide sequence. For this reason, discrimination tests based on competitive binding of probes can usually only be used for relative frequency estimation of a variant and not for absolute quantification

Examples of the results for all three types of assays are shown in Fig. 3. When shown in a one-dimensional plot (i.e. fluorescence is evaluated for a target sequence), each dot represents either a positive (here in blue) or negative (in grey) droplet for the target sequence. These droplets are distinguished as positive or negative by manually setting a threshold (on the *y*-axis) in arbitrary fluorescence amplitude units (AU). Typically, the threshold is determined by including both the negative and positive control samples in the initial optimisation run. In our experience, this need not be done for each subsequent run.

When sufficient quality of sample DNA (in terms of fragment length and integrity) is achieved and reaction conditions have been optimised, a clear separation between the different droplet populations is usually seen, with little to no "rain", i.e. trailing droplets, observed between the positive and negative droplets. Interpreting the results of duplex and discrimination tests is a little more complex. This is because some droplets may contain both target sequences, while others may contain only one or none at all. Thus, if two differently labelled hydrolysis probes are used, there can be up to four different droplet populations. However, as can be seen in Figs. 3B and C, these can be conveniently separated and displayed (with two adjustable thresholds) in a two-dimensional plot. Perhaps most importantly, the analysis software helps the user by trying to predict the thresholds (between positive and negative droplets) automatically. In each case, copy numbers and fractional abundance estimates are calculated for each target amplicon sequence and are available to the user immediately. Currently, the latest technology is the Bio-Rad QX600 Droplet Digital PCR System, which allows the simultaneous use of up to six fluorophores, enabling the quantification of up to 12 targets in a single well.

### Applications

**Fig. 4:**
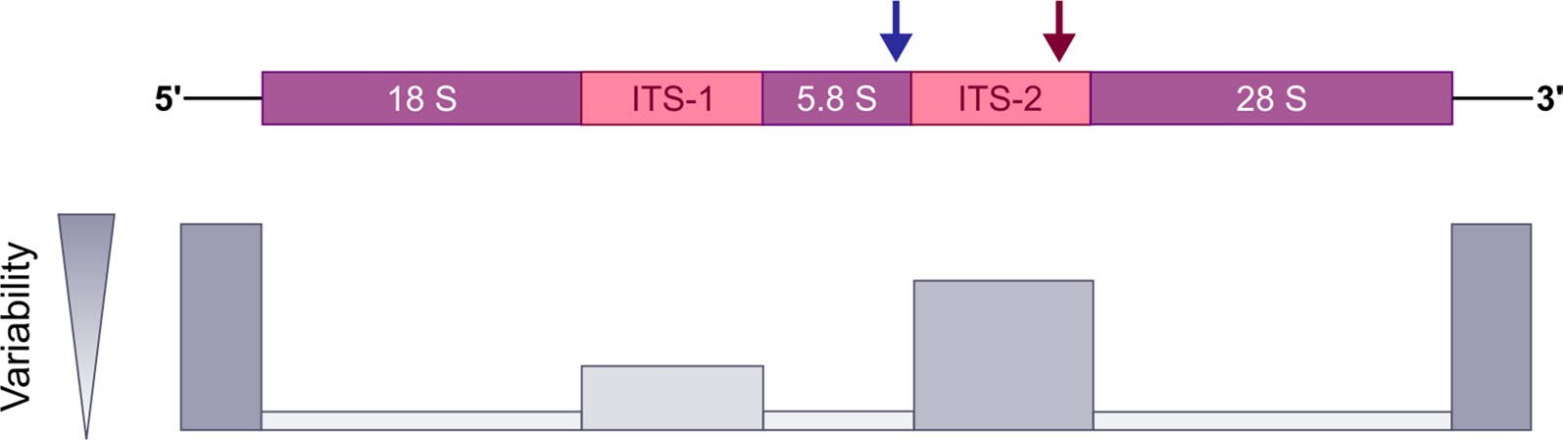
Schematic representation of part of the ribosomal nuclear DNA (rDNA) gene cluster and the variability of the different loci. This example shows two DNA targets (terminal 3′ end of 5.8 S and terminal 3′ end of ITS2) used to quantify the three major strongylid nematodes in the gastrointestinal tract of sheep. The blue arrow indicates the position for which a universal primer and probe pair is used to quantify all strongylid nematode species, while the red arrow indicates the position in ITS2 where a different primer and probe pair quantifies the specific nematode genera present in a sample

In the following (sub)sections, we review some of how dPCR can be used to quantify parasites, using samples from the hosts or their environment. Although dPCR technology can be used to detect and quantify any reverse-transcribed RNA or DNA sequence, loci in ribosomal DNA (rDNA) clusters are commonly used in diagnostic tests for parasites [16]. There are several reasons for this, the most important of which is that rDNA clusters are present in tandem repeats throughout the eukaryotic genome. Second, each rDNA gene cluster contains both the conserved (coding for 18S, 5.8S and 28S) and the more variable, internal transcribed spacer (ITS), regions (Fig. 4). These unique features of the rDNA gene cluster allow the design of both universal and unique primer–probe sets, each targeting the major taxonomic rank such as family, genus or species. Third, rDNA sequences have been well studied over the years, especially in parasites of medical and veterinary importance, providing an extensive, publicly available reference sequence dataset that characterises the most common species. Nevertheless, regions of mitochondrial DNA (mtDNA) as well as other loci in the genomic DNA of both helminths and protozoa are suitable targets for parasite detection [17]. For the detection of mutations conferring resistance to antiparasitic drugs, the causal (or closely related) variants must be known a priori.

### Uniplex assays

Uniplex assays have been used mainly for the diagnosis of parasitic worms and protozoa, using either TaqMan hydrolysis probes or DNA-binding dyes. For example, researchers in China developed a ddPCR protocol for the detection of the ITS sequence belonging to the whipworms *Trichuris* spp. (transmitted through the soil), which are responsible for enteritis in a variety of mammalian hosts [18]. This assay also proved to be highly specific (i.e. it resulted in negative amplification outcomes when tested on other parasite species from the same hosts) and was able to reliably estimate low target copy numbers. In addition, a *Schistosoma* assay targeting a sequence fragment of mitochondrial *nad1* was developed, and the amplified fragment was detected using the EvaGreen^®^ intercalating dye [19]. After testing on four different human body fluids, the authors found the faecal samples to be a valuable source of parasite material for surveillance in areas where schistosomiasis incidence (or re-occurrence) is a concern. Similarly, probe-based *Echinococcus multilocularis* assays were used on > 100 liver samples from rodent intermediate hosts, most of which had no macroscopic lesions [20]. When comparing the ddPCR and qPCR results, it was found that the latter underestimated the exposure pressure. Similarly, a uniplex *Toxoplasma gondii* detection assay performed better than qPCR when testing the diaphragm tissue samples from farm animals [21].

Furthermore, the characteristics of the assays for absolute quantification of haemosporidian parasites in birds (*Plasmodium*, *Haemoproteus* and *Leucocytozoon*) were compared with those of other assays (qPCR and standard nested PCR) using blood samples from birds of prey [22]. Especially for low-intensity samples, the newly developed ddPCR protocol provided more consistent and accurate copy measurements compared to the then widely used general qPCR assay. Similarly, a probe-based ddPCR assay was developed for the absolute quantification of *cox3* in the tick-borne apicomplexan parasite *Cytauxzoon felis*, which causes severe disease in cats [23]. As the performance of this assay was improved compared to the then existing PCR assay, it was considered more useful for monitoring treatment efficacy and detecting the relapse of infection. Thus, the traditional advantages of ddPCR, particularly its higher sensitivity, precision and accuracy, especially when working with small parasite populations (low intensity of infection), remain unchallenged by other similar amplification-based technologies.

Other less conventional applications of the duplex assay approach in parasitology include, for example, the study of gene expression changes associated with in vitro development of the heartworm *Dirofilaria immitis* microfilariae [34]. The authors in this study were able to demonstrate that morphologically different larvae have different expression levels of target genes involved in the ecdysone signalling cascade.

### Duplex assays

Duplex assays (or multiplex assays when more than two primer–probe pairs are used) are well suited for studying the relative proportion of a particular parasite in a mixed-species sample, especially when the morphological characteristics of several species overlap (so that they are visually indistinguishable from each other). In a study on parasites in sheep, sequence variation in four different loci of the rDNA gene array was used to quantify the major strongyle nematodes infecting the gastrointestinal tract [24]. For one of these highly conserved regions, a universal primer–probe set was designed to detect all major strongylid species, while the other three were targeted to specific parasite genera (*Haemonchus*, *Teladorsagia* and *Trichostrongylus*). Multiplexing made it possible to quantify each parasite genus in coproculture containing mixed parasite populations. In addition to the obvious diagnostic utility, the results of the above study suggest that this (and similar) assay(s) is a useful adjunct to applications that rely on conventional egg-counting methods for the detection of anthelmintic resistance (i.e. the faecal egg reduction test). Furthermore, the *Haemonchus*-specific assay has been used not only to confirm the lack of efficacy of monepantel against *H. contortus* [25] but also to show that this species is closely associated with resistance to the drugs most commonly used in sheep farms (ivermectin and benzimidazoles) [26]. The *Haemonchus*-specific assay was also integrated into an extended sampling protocol to quantify this highly pathogenic parasite in Swedish sheep farms [27] and used in a study on the dynamics of periparturient increase in winter and spring lambing ewes [28]. Later, a similar approach was used for the simultaneous identification and absolute quantification of ascarid nematode eggs in laying hens (*Ascaridia galli* and *Heterakis gallinarum*). In this application, the assay was based on two different genus-specific primer–probe sets targeting the ITS2 regions of the two parasites [29]. Not only could both parasites be detected with ddPCR, but a 6% higher detection rate was also achieved compared to a flotation-based method.

Duplex assays have also been used to study protozoan parasites. For example, in one study, absolute quantification of oocysts from seven *Eimeria* spp. was performed using hydrolysis probes targeting the *cox3* gene sequence and another nearby rDNA fragment [30]. Similarly, four novel ddPCR duplex assays were tested on *Plasmodium falciparum* carrying different deletions in genes encoding histidine-rich proteins (*hrp2* and *hrp3*) secreted during parasite replication [31]. The authors of the said study concluded that the assay targeting *hrp2* deletion will facilitate molecular surveillance and thus aid in the selection of diagnostic tests to accelerate the control and elimination of malaria. Similarly, duplex reaction assays were developed for the quantification of 18S ribosomal RNA (rRNA) at the genus and species level in four human *Plasmodium* species [32]. In general, these assays showed higher sensitivity than qPCR in detecting very low parasitemia of all species when tested on blood samples. In addition, a probe-based multiplex assay was optimised for the simultaneous detection of a broad range of different pathogens, including different *Babesia* species, and validated on a variety of clinical samples from animals and humans [33].

In addition, a duplex assay to determine the relative abundance of a 63-bp deletion in *hco-acr-8* in *H. contortus*, at the time thought to be associated with levamisole resistance in ovine nematodes, was proposed as an anthelmintic resistance screening tool [35]. In this study, the authors used two primer–probe pairs, one for a reference locus in exon 1 and the other for the deletion-containing locus in intron 2, to estimate the overall relative abundance of the deletion in individual adult-stage parasites and mixed larval populations. Similarly, a duplex assay was developed and validated for highly sensitive monitoring of *Plasmodium* and insecticide resistance mutations in the malaria mosquito vector [36]. In summary, the use of multiplexing in ddPCR assays enables a wide range of different applications aimed at the simultaneous detection and quantification of multiple DNA targets.

### Discrimination assays

Discrimination assays represent a powerful, versatile approach for detecting mutations or genetic variations associated with a particular phenotypic trait, such as drug resistance, or even a species. Several recent studies have used the discrimination assay approach to detect mutations associated with drug resistance (especially in nematode parasites of animals). The first study was designed to quantify the frequency of the mutant allele in a gene encoding the dye-filling protein (*dyf-7*), which was then thought to be associated with ivermectin resistance in *H. contortus* [37]. Several other complementary assays have been developed for phenotypic characterisation of *H. contortus* isolates as either resistant or sensitive to benzimidazole drugs based on the presence (or absence) and frequency of benzimidazole resistance-mediating mutations in isotype 1 *β-tubulin* [38–40]. In the aforementioned studies, the authors compared mutation frequency results from dPCR with different sequencing platforms to establish consistency between the different methods.

An example of a discrimination test to distinguish between parasite species is a study conducted on two cattle nematodes [41]. Here, the authors used a single universal primer pair and two competing hydrolysis probes to estimate the abundance of parasites of the genera *Cooperia* and *Ostertagia*, and an additional universal probe to allow robust and accurate quantification of both species. In summary, discrimination tests based on the competitive binding of probes allow the development of unique approaches to investigate the exact proportion of multiple sequence variants, whether SNPs or indels, in the selected samples.

### Environmental DNA

Digital PCR technology has also been used to detect parasites and/or their hosts from (collected by non-invasive means) environmental samples and is a powerful method to assess the presence of species in space and time in both aquatic and terrestrial environments. One of the earliest examples of a study using both environmental (e)DNA and dPCR is the series of experiments conducted by researchers in Australia [42]. In this study, however, the authors used dPCR as a complementary (rather than primary) tool to quantitatively validate positive control samples containing the protozoan parasite *Chilodonella hexasticha*. A few years later, Norwegian researchers attempted to detect eDNA of the extremely pathogenic (to Atlantic salmon) *Gyrodactylis salaris* using ddPCR as the primary method for analysis and quantification [43]. In their study, the authors used a duplex assay to simultaneously detect both the parasite (using the ITS1 locus) and the host species (cytochrome b locus) in filtered water. As eDNA detection is less labour-intensive and eliminates the need to sacrifice live fish, the authors suggested that the newly introduced method could complement or even replace traditional techniques for monitoring natural freshwater systems for the risk of *Gyrodactylis salaris* transmission.

Another example of screening environmental samples for the presence of parasite DNA is a uniplex protocol for screening *Taenia solium* DNA in soil [44, 45]. The proposed assay was developed and validated using a primer–probe set targeting the mitochondrial *cox1* sequence and subsequently used as a screening tool in pig-keeping households in rural villages in central Tanzania. Finally, a very recent study proposed a multiplex ddPCR assay targeting short sequence fragments in the ITS2 region of the helminth parasite *Fasciola hepatica* and its intermediate host *Galba truncatula*, using eDNA (filtered water samples collected from pastures with grazing animals) as template input [46]*.* Perhaps unsurprisingly at this point, the multiplex test used by the authors had higher sensitivity, identifying more samples positive for *G. truncatula* and *F. hepatica* compared with similar methods. In conclusion, monitoring both parasites and their hosts using eDNA and dPCR is a powerful approach for studying parasite-host interactions in different ecosystems, as well as for monitoring the effectiveness of management strategies aimed at limiting the suitability of habitats for different aquatic or soil-borne parasites and their intermediate hosts.

### Statement

With sequencing technologies cheaper and more available than ever, and the rapidly growing number of reference genomes and computational resources, we are entering the age of genomics to answer complicated questions about parasite biology and transmission. As we begin to decipher complicated traits and identify the genetic variants responsible for them, a diagnostic platform to quantitatively screen populations for the presence and abundance of these traits will be paramount. dPCR has an attractive sensitivity, specificity and precision profile (among other advantageous features), making it a robust candidate for future studies to quantify genetic variants associated with various traits in parasitic organisms. Recent developments also point to another new avenue for the development of ddPCR assays—the study of epigenetic changes in genomic DNA [47]. Thus, ddPCR is a truly versatile tool in molecular parasitology, with a wide range of applications to study not only parasites, their ecology and interactions with hosts, but also sequence variants associated with different phenotypic traits or species.

## Conclusions

In this review, we have discussed several different applications of dPCR for the detection of helminths or protozoa in a variety of diagnostic samples. In addition to the detection of parasites, several dPCR-based assays have been optimised for the detection of genetic variations associated with specific phenotypic traits (here drug resistance) in parasites. The use of dPCR offers unique opportunities for the rapid identification of parasite DNA with unparalleled precision using a variety of samples from different hosts and/or the environment. The main advantages compared to other amplification-based technologies are as follows: (i) dPCR provides accurate absolute quantification (especially at low copy numbers), (ii) does not require downstream calculations by the user and iii) is also less susceptible to PCR inhibitors as well as variations in amplification efficiency. Finally, the overwhelming number of studies suggests that the reproducibility, precision and accuracy of ddPCR assays exceed those of similar methods (e.g. qPCR).

## Data Availability

Data sharing is not applicable to this article as no datasets were generated or analysed during the current study.

## Abbreviations

*acr*: Acetylcholine receptor genes
AU: Amplitude units
*cytb*: Cytochrome b genes
dPCR: Digital PCR
ddPCR: Digital droplet PCR
*dyf*: Dye-filling protein genes
ITS: Internal transcribed spacer
*hrp*: Histidine-rich protein genes
mtDNA: Mitochondrial DNA
*nad1*: NADH dehydrogenase subunit 1 gene
PCR: Polymerase chain reaction
qPCR: Quantitative real-time PCR
RNA: Ribonucleic acid
rRNA: Ribosomal RNA
SSU: Small subunit
